# Oxidation and Polymerization of Triacylglycerols: In-Depth Investigations towards the Impact of Heating Profiles

**DOI:** 10.3390/foods8100475

**Published:** 2019-10-11

**Authors:** Yih Phing Khor, Khai Shin Hew, Faridah Abas, Oi Ming Lai, Ling Zhi Cheong, Imededdine Arbi Nehdi, Hassen Mohamed Sbihi, Mohamed Mossad Gewik, Chin Ping Tan

**Affiliations:** 1 Department of Food Technology, Faculty of Food Science and Technology, Universiti Putra Malaysia, UPM Serdang, 43400 Selangor, Malaysia; sweet_appie@hotmail.com (Y.P.K.); khaishin92@gmail.com (K.S.H.); 2 Department of Food Sciences, Faculty of Food Science and Technology, Universiti Putra Malaysia, UPM Serdang, 43400 Selangor, Malaysia; faridah_abas@upm.edu.my; 3 Department of Bioprocess Technology, Faculty of Biotechnology and Molecular Sciences, Universiti Putra Malaysia, UPM Serdang, 43400 Selangor, Malaysia; omlai@upm.edu.my; 4 Department of Food Safety and Quality, School of Marine Science, Ningbo University, Ningbo 315211, China; lingzhicheong@yahoo.com; 5 Chemistry Department, College of Science, King Saud University, P.O. BOX 2455, Riyadh 11451, Saudi Arabia; imed12002@gmail.com (I.A.N.); hmsbihi@ksu.edu.sa (H.M.S.); mmossad@ksu.edu.sa (M.M.G.); 6 Chemistry Department, El Manar Preparatory Institute for Engineering Studies, Tunis El Manar University, P.O. Box 244, Tunis 2092, Tunisia

**Keywords:** monomeric oxidized triacylglycerol, polymerized triacylglycerol, epoxy acid, keto acid, hydroxy acid, palm olein, oil oxidation, polymerization, oxidative stability, total polar compound

## Abstract

The stability of refined, bleached, and deodorized palm olein (RBDPO) was studied under controlled heating conditions. RBDPO was heated continuously for 24 h at 160, 170, and 180 °C, with oil sampled at four hour intervals. Thermo-oxidative alterations were measured through various parameters, such as monomeric oxidized triacylglycerols (oxTAG), total polar compounds (TPC), polymerized triacylglycerols (PTG), oxidative stability, and fatty acid composition. After 24 h of heating, the TPC and triacylglycerol oligomers showed a linear increase with heating time at all heating temperatures. At the end of the heating study, more epoxy acids were formed than keto and hydroxy acids. Moreover, caprylic acid, which was not present in fresh oil, was formed in significant amounts. The increase in oxTAG was strongly correlated with the increase in the *p-*anisidine value and total oxidation value. The decreases in diacylglycerol and free fatty acids were strongly correlated with an increase in PTG.

## 1. Introduction

Oil undergoes hydrolysis, oxidation, and polymerization during heat treatment, resulting in the formation of total polar compounds (TPC) that have a higher molecular weight and higher polarity than normal unaltered triacylglycerols (TAG). These polar fractions can be isolated from the oil, and the level of the polymerized triacylglycerol (PTG) distribution is considered to be the most reliable quality index for oil [1]. It is noteworthy that oil samples sharing a similar TPC content may exhibit different PTG distributions. Furthermore, palm olein naturally contains a higher diacylglycerol (DAG) content, and this eventually leads to a higher TPC value in fresh palm olein compared to the other types of oil. Therefore, it is important to understand the contribution of each PTG compound to the total alteration of the oil when it is subjected to high heat. During high performance size exclusion chromatography (HPSEC) analysis, each PTG peak illustrated in the chromatogram corresponds to a complex group of substances. Oxidized compounds can be present in hundreds or even thousands of combinations depending on the fatty acyl groups that esterify on the TAG molecule. Therefore, the quantitative determination of each specific compound is relatively difficult. 

Monomeric oxidized triacylglycerols (oxTAG) are formed during processes involving fats and oils in the presence of oxygen via oxidative degradation. These molecules have gained research interest among the complex mixture of oxidation products formed due to their high absorbability [2]. More oxTAG is formed during processes at high temperatures, while minor amounts may be formed during the storage of fats and oils [3]. The determination of oxTAG levels can provide an initial indication about whether used frying fats and oils are still fit for consumption. The fatty acid composition, heating temperature, and production of the oil are a few important factors that affect the formation of oxTAG [4].

Both PTG and oxTAG are associated with the development of atherosclerosis, liver damage, diabetes, and the promotion of intestinal tumors [5]. Various research studies have been conducted on highly unsaturated oils such as sunflower, soybean, olive, linseed, rapeseed, and camellia seed oils [1,4,6,7] to determine their PTG or oxTAG changes and their formation during heating treatments. However, literature is scarce regarding both the oxTAG and PTG changes together in refined, bleached, and deodorized palm olein (RBDPO) when it is subjected to high temperature continuously for a period of time and using a without-food system. It is vital to understand the extent of overall oxidative deteriorations in RBDPO in a fundamental study to provide more insights for other related works in the future. 

On the other hand, the formation of short-chain fatty acid such as caprylic acid when the oil is subjected to elevated temperatures has also been of great interest. A review showed that short-chain fatty acid was a product resulting from the breakdown of fatty acid hydroperoxides or oxidation processes [8]. The quantification of short-chain fatty acid would be able to provide a good indication of the total alteration level in used frying oil. However, this analysis is only suitable to be conducted on a sample that did not originally contain any short chain fatty acid. To date, there no legal limit has been set for the presence of short-chain fatty acid resulting from oil degradation. Furthermore, there is a lack of information regarding the relationship between oxTAG, PTG, and caprylic acid formation, which is worth investigating. 

The objective of the present study was to evaluate the oxidation and structural decomposition of RBDPO using a controlled heating system. The stability of the frying oil plays a vital role, as it influences the quality of fried products [9]. Palm olein, the liquid fraction obtained during the fractionation of palm oil, is commonly used as a frying medium [10], especially in some of the 24 h-operating fast food chains whereby the oil is continuously heated. Following this experiment, it will be possible to gauge the quality of oil when it is heated for a period of time. 

## 2. Materials and Methods 

### 2.1. Chemicals

Methyl heneicosanoate (C21:0), methyl 12-hydroxystearate, and methyl 12-oxostearate were purchased from Sigma-Aldrich (Darmstadt, Germany). Methyl *trans*-9,10-epoxystearate was purchased from Santa Cruz Biotechnology, Inc. (Dallas, TX, USA). Silica gel 60 (particle size = 0.063−0.200 mm) for column chromatography was acquired from Merck (Darmstadt, Germany). Platinum (IV) oxide hydrate was purchased from Thermo Fisher Scientific (Waltham, MA, USA). Except tetrahydrofuran, which was used as the mobile phase, was of HPLC grade. All of the solvents and chemicals used for the analyses were of analytical grade.

### 2.2. Materials 

Refined, bleached, and deodorized palm olein (RBDPO) (iodine value 56) without the addition of antioxidants was purchased from Moi Foods Malaysia Sdn. Bhd. (Selangor, Malaysia).

### 2.3. Samples

Refined, bleached, and deodorized palm olein (RBDPO) was studied under controlled heating conditions. RBDPO (3.5 L) was heated continuously for 24 h in a Cornell batch fryer (Petaling Jaya, Malaysia) at 160, 170, and 180 °C, with 100 mL of oil sampled at 4-h intervals. The fryer was left uncovered during the heating process. The oil samples were stored at −20 °C for further analyses. The heating experiments were conducted in replicates and without any oil replenishment. 

**Figure 1:**
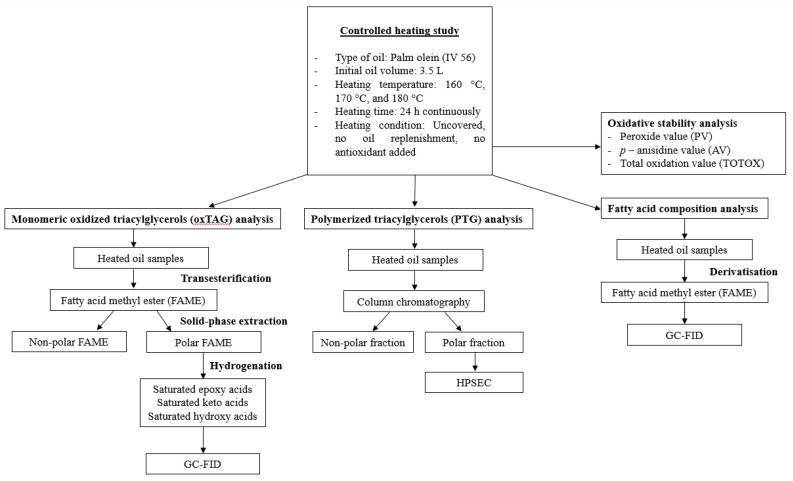
Flowchart of the experimental design. HPSEC: high performance size exclusion chromatography; GC-FID: gas chromatograph equipped with a flame ionization detector.

The detailed experimental design is illustrated in Figure 1.

### 2.4. Determination of Epoxy, Keto, and Hydroxy Fatty Acids

#### 2.4.1. Transesterification

Derivatization using sodium methoxide was conducted at room temperature to produce fatty acid methyl esters (FAMEs). A 300 mg oil sample was weighed into a 15 mL centrifuge tube, and 3 mL of *tert*-butyl methyl ether (TBME) was added. This was followed by the addition of 1.5 mL of 0.2 M sodium methoxide in methanol. The tube was screw-capped, vortexed for 1 min, and allowed to stand at room temperature for 2 min. Then, 0.1 mL of 0.5 M sulfuric acid was added to neutralize the mixture, and the tube was vortexed for 5 s. Next, 3 mL of ultrapure water was added, vortexed for 10 s, and centrifuged at 4000 rpm for 5 min. The supernatant was transferred into a separate vial and evaporated to dryness under nitrogen [7].

#### 2.4.2. Isolation of Polar FAMEs

The fractionation of FAMEs was conducted using solid-phase extraction (SPE) to separate them into polar and non-polar FAMEs. The SPE analysis was produced using a vacuum manifold. A silica-based SPE cartridge with 1 g of sorbent was chosen for the analysis. The SPE cartridge was pre-conditioned before use. A total 100 mg of FAMEs was accurately weighed, dissolved in 2 mL of n-hexane-diethyl ether (98:2; *v/v*), and transferred into the SPE cartridge. The non-polar fraction was eluted with 15 mL of n-hexane-diethyl ether (98:2; *v/v*). Next, the polar fraction was eluted with 25 mL of diethyl ether. For an internal standard, 1 mL of C21:0 (500 µg/mL) dissolved in TBME solution was added to the collected polar fraction, and the solvent was evaporated under nitrogen [7]. 

#### 2.4.3. Hydrogenation

The polar fraction, which was composed of the altered FAMEs, was dissolved in 2 mL of methanol. Platinum (IV) oxide hydrate was used as the metal catalyst (Adam’s catalyst) for the hydrogenation process. Hydrogenation was carried out by bubbling hydrogen into the sample at room temperature for 10 min. Finally, the methanol was evaporated under nitrogen. The sample was dissolved in 1.5 mL of diethyl ether, and then it was filtered. The sample was then ready for injection into a gas chromatograph equipped with a flame ionization detector (GC-FID) [7].

#### 2.4.4. Gas Chromatography

Monomeric oxidized triacylglycerols were dissolved in diethyl ether and analyzed by gas chromatography using an Agilent 6890 Series chromatograph (Agilent Technologies, Santa Clara, CA, USA) equipped with a split-splitless injector operating in the split mode with a 20:1 split ratio at 250 °C, a J&W DB-Wax fused-silica capillary column, 60 m × 0.25 mm (internal diameter), film thickness of 0.25 µm (J&W Scientific, Folsom, CA, USA) and a flame ionization detector that was used at 250 °C with hydrogen at 40 mL/min, air at 450 mL/min, and nitrogen as the auxiliary gas at 45 mL/min. The analyses were run using nitrogen as the carrier gas at 1 mL/min and under isothermal conditions of 240 °C for 30 min [7]. The signal-to-noise ratios of each peak were monitored to ensure the reliability of the quantified data. The monomeric oxidized triacylglycerols contents were determined from the calibration curve constructed by plotting the concentration of standards versus the corresponding peak areas.

### 2.5. Determination of Polymerized Triacylglycerols

#### 2.5.1. Isolation of Total Polar Compound

The content of polar compounds was determined by a combination of adsorption and size-exclusion chromatography [3]. Non-polar and polar fractions were separated into two fractions by silica column chromatography. A 40 × 1 cm I.D. glass column filled with 10 g of silica adjusted to a water content of 5% (*w/w*) was used. First, the non-polar fraction, which comprised the unaltered TAGs, was eluted with 75 mL of n-hexane-diethyl ether (90:10, *v/v*). Then, the polar fraction was eluted with 75 mL of diethyl ether. The solvent was evaporated using a rotary evaporator under reduced pressure. After the evaporation of the solvents, the polar fraction was accurately weighed and calculated. The polar fraction was dissolved in tetrahydrofuran (THF) to achieve a concentration of 50 mg/mL [7]. The sample was then ready for injection into a high performance size-exclusion chromatograph with a refractive index detector (HPLC-SEC/RI). 

#### 2.5.2. High-Performance Size-Exclusion Chromatography (HPSEC)

The polar fraction of oil was analyzed by HPSEC to determine the contents of oligomers, dimers, monomers, DAG, and free fatty acids (FFA). The polar fraction of the oil was dissolved in tetrahydrofuran and analyzed using a Shimadzu HPLC chromatograph equipped with a SIL-10AD injector, a LC-20AD pump, and a RID-10A refractive index detector. The separation was performed on two 100 and 500 Å phenogel columns (30 × 0.78 cm internal diameter) packed with porous, highly cross-linked styrene–divinyl benzene copolymers (film thickness 5 µm) (Phenomenex, Torrance, CA, USA) connected in series, with tetrahydrofuran (1 mL/min) as the mobile phase. The columns were connected in a series to improve the separation of the oil components. Each group of the polar fraction distributions was quantified using the following equations:

P_oligomer_ = (A_oligomer_/∑A)·P_TPC_ (1)

P_dimer_ = (A_dimer_/∑A)·P_TPC_ (2)

P_oxTAG_ = (A_oxTAG_/∑A)·P_TPC_ (3)

P_DAG_ = (A_DAG_/∑A)·P_TPC_ (4)

P_FFA_ = (A_FFA_/∑A)·P_TPC_ (5)

where P_TPC_, P_oligomer_, P_dimer_, P_oxTAG_, P_DAG_, and P_FFA_ represent the percentages of total polar compound, TAG oligomers, TAG dimers, monomeric oxidized triacylglycerols, diacylglycerols, and free fatty acids found in the polar fraction of the oil sample, respectively; A_oligomer_, A_dimer_, A_oxTAG_, A_DAG_, and A_FFA_ represent the peak area of each specific fractions; and ∑A represents the sum of all peak areas [7].

### 2.6. Determination of Oxidative Stability

#### Peroxide Value (PV), *p*-Anisidine Value (AV), and Total Oxidation (TOTOX) Value

The peroxide value [11] and the *p*-anisidine value [12] of the oil samples were determined according to The American Oil Chemists’ Society Official methods (method Cd 8b-90 and Cd 18-90). Total oxidation (TOTOX) value was calculated using the following formula:

TOTOX = 2PV + AV(6)

### 2.7. Determination of Fatty Acid Composition

#### 2.7.1. Derivatization

Derivatization to FAMEs was carried out using a base-catalyzed method at room temperature to avoid the losses of volatile FAMEs. A total of 100 mg of each of the oil samples was accurately weighed into a centrifuge tube, and then 2 mL of hexane containing 1 mg/mL methyl tridecanoate (C13:0) was used to dissolve the oil sample. Next, 0.1 mL of 2 M potassium hydroxide in methanol was added. The tube was screw-capped and vortexed for 15 s. The mixture was centrifuged at 4000 rpm for 5 min. The organic layer was filtered using 0.22 µm PTFE-type syringe filter and was then ready for injection into a gas chromatograph equipped with a flame ionization detector (GC-FID) [13].

#### 2.7.2. Gas Chromatography

The FAME was analyzed by gas chromatography using an Agilent 6890 Series chromatograph (Agilent Technologies, Santa Clara, CA, USA) equipped with a split-splitless injector operating in split mode with a 20:1 split ratio at 250 °C, a SGE BPX70 column, 25 m × 0.32 mm internal diameter, a film thickness of 0.25 µm (Trajan Scientific, Australia), and a flame ionization detector at 280 °C with hydrogen at 40 mL/min, air at 450 mL/min, and nitrogen as the auxiliary gas at 25 mL/min. The analyses were run using nitrogen as the carrier gas at 10 psi. The oven temperature was held for 5 min at 100 °C, followed by an increment of 4 °C/min up to 240 °C, and then held for 20 min. A calibration curve for caprylic acid (*R*^2^ = 0.99) was separately constructed to quantify its concentration. 

### 2.8. Statistical Analysis

All the heating experiments and all analyses were conducted in duplicate. The experimental results were analyzed using Minitab software (Minitab Version 17.1, Minitab Pty Ltd., Sydney, New South Wales, Australia). All measurement data were expressed as the mean values ± standard deviations. A one-way analysis of variance (ANOVA) and post-hoc Tukey’s test with a 5% significance level was used to detect significant differences (*p* < 0.05) between the mean values. A Pearson correlation coefficient was used to examine the relationships among all analytical parameters. 

## 3. Results and Discussion

### 3.1. Effect of Temperature and Heating Time on the Formation of Monomeric Oxidized Triacylglycerols

Monomeric oxidized triacylglycerols (oxTAG) are characterized by the presence of an extra oxygen in at least one of the fatty acyl chains of the molecule. oxTAGs are formed from the breakdown or decomposition of primary oxidation products such as hydroperoxides. These secondary oxidation products that accumulate in thermoxidized oils are relatively stable [7]. The oxTAGs have been identified as a mixture of epoxy, keto, and hydroxy. A methylation step followed by the separation of polar and nonpolar FAME by solid phase extraction prior to gas chromatography analysis was conducted to eliminate other interfering fatty acids in the oil such as non-altered fatty acids [7,14]. Hydrogenation was conducted in order to reduce the number of double bonds, simplify the structures of the compounds, and subsequently reduce the number of analytes [7]. 

**Figure 2:**
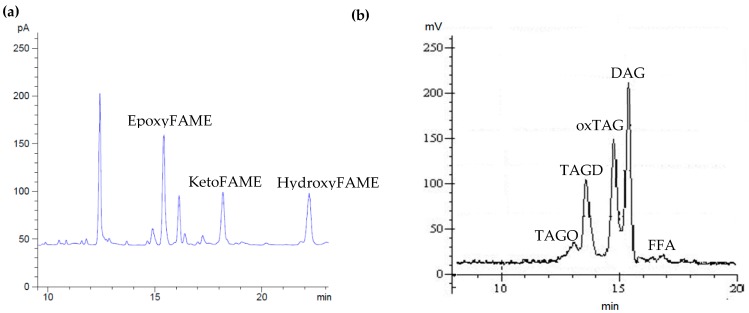
(**a**) Sample gas chromatograph (GC) chromatogram for the determination of epoxy, keto, and hydroxy acids and (**b**) sample high performance size exclusion chromatography (HPSEC) chromatogram for the determination of polar fraction distribution (TAGO: Triacylglycerol oligomer; TAGD: Triacylglycerol dimer; oxTAG: Monomeric oxidized triacylglycerol; DAG: Diacylglycerol; and FFA: Free fatty acids).

The elution order of the oxTAGs began with saturated epoxy, followed by keto and hydroxy acids, an order which is consistent with their polarity (Figure 2) [7]. These oxTAGs were not present in the fresh RBDPO but they increased significantly (*p* < 0.05) with the temperature and heating time (Table 1). At the end of the heating times at 160, 170, and 180 °C, higher epoxy acids were formed than the keto and hydroxy acids. The abundance of oleic and linoleic acid in palm olein explained the formation of more epoxy acids than keto and hydroxy acids. This is because methyl epoxystearates were formed from methyl oleate and methyl epoxyoleates from methyl linoleate [7]. 

A previous study reported that oxTAG ranged from 5.9% to 9.4% in used frying fats and oils that reached 25% polar compounds (the limit for human consumption) [3]. However, that study mainly focused on soft oils, which are commonly used in European countries. In contrast, in this study on the heating of palm olein, the highest total oxTAG was 1.5% when palm olein was heated at 180 °C for 24 h. Official food control in Europe reported that 7 g/kg epoxy fatty acids was set as the limit for usability of used frying fats and oils [4]. In this case, palm olein heated at 180 °C exceeded the safe limit, as the epoxy acids concentration increased to 7.3 g/kg after being heated for 24 h. The total epoxy, keto, and hydroxy fatty acids contributed 8.8–15 g/kg in RBDPO at the end of 24 h of heating. In this study, the influence of heating time and temperature towards the formation of oxTAG was significant (*p* < 0.05).

### 3.2. Effect of Temperature and Heating Time on the Total Polar Content (TPC)

Total polar content (TPC) is one of the most commonly used parameters to evaluate the degradation or quality of oil [15,16]. The limit of rejection for used cooking oil has been set at 25% in most European countries. A previous study reported that the formation of polar compounds is strongly related to the primary and secondary oxidation processes that occur in the oil during heating or frying [16]. In this study, the total polar content (TPC) increased linearly (*R*^2^ = 0.99) with heating time at all heating temperatures (Table 2). TPC in RBDPO exceeded 25% after heating for 24, 20, and 16 h at 160, 170, and 180 °C, respectively. The rate of degradation was highest in RBDPO heated at 180 °C for 24 h because TPC increased by 293% compared with the fresh RBDPO. On the other hand, the percentage of TPC increment was 199% and 262% in RBDPO heated at 160 and 170 °C, respectively, for 24 h. In other words, the extent of degradation in RBDPO heated at 180 °C was 1.45 times faster compared with RBDPO heated at 160 °C. Bansal et al. (2010) showed that TPC reached 32% in palm olein after heating for 24 h at 180 °C. In their experiment, palm olein was continuously heated six hours daily for four days. Our study showed a higher TPC (33%) when RBDPO was heated at 180 °C continuously for 24 h. This demonstrated that the rate of oil deterioration was faster when the oil is heated continuously rather than intermittently without oil replenishment. 

### 3.3. Effect of Temperature and Heating Time on the Formation of Polymerized Triacylglycerols

The evaluation of polymerized triacylglycerol (PTG) formation in oil is the most reliable measure of the extent of oxidative degradation. Measuring this group of compounds is of paramount importance because it not only detects the oxidized products formed before rancidity occurs (during initial stage) but also gauges the oxidation of oil at later stages [17]. Furthermore, because the amount of diacylglycerols (DAG) is high in palm olein compared with other oils and it is quantified as one of the fractions of TPC, it is less accurate to judge oil quality based solely on TPC. Therefore, the determination of PTG together with TPC and DAC is more representative in this case to gauge the quality of oil, especially the used oil [15]. The sample HPSEC chromatogram is illustrated in Figure 2.

In this study, a significant change in PTGs was observed, which indicated that heating induced the breakage and reformation of TAG. TAG oligomers, TAG dimers, and oxidized TAG monomers increased significantly (*p* < 0.05) with heating time (Table 2). The limit for the rejection of a cooking oil for PTG ranged between 12% and 13%, with 10% PTG recommended as the most appropriate level for the rejection for oil replenishment [18]. Because there was no oil replenishment in this study, the PTG concentration already exceeded 12% when RBDPO was heated for 24 h at 170 °C and 20 h at 180 °C. At the same time, the TPC already exceeded the 25% limit of rejection. A previous study suggested that at high temperature (>140 °C), hydroperoxides decompose and lead to a polymerization reaction and the formation of dimers [6,19]. A significant (*p* < 0.05) increase in polymers denotes the onset of an advanced oxidation stage [20].

As shown in Table 2, it is clear that when TPC achieved 25%, which is the limit of oil rejection, the PTG content formed in palm olein was approximately 10%. It is essential to note that the TAG oligomer content increased linearly (*R*^2^ = 0.98 at 160, 170, and 180 °C) with heating time and temperature. The TAG oligomer was a new polar compound formed upon heating in RBDPO, as it was not detected in the fresh oil. At the end of the heating study, 6.74% of the TAG oligomer was found in RBDPO heated at 180 °C for 24 h. In other words, it was approximately 85.16% and 24.81% higher than the RBDPO heated at 160 and 170 °C, respectively. After 20 h in RBDPO heated at 160, 170, and 180 °C, there were no significant increase (*p* > 0.05) in the TAG dimer content. This was attributed to the simultaneous conversion of some of the dimers into oligomers and thermal degradation [21]. 

As TAG is nonpolar and was already removed by silica gel chromatography, the monomer species detected in the polar fractions contained only the polar monomeric oxidized triacylglycerols [22]. A previous study reported that, similar to oxTAG, the formation of PTG was affected by the degree of oil unsaturation, because more unsaturated oils are susceptible to polymerization [18]. Unlike PTG, there were no significant changes (*p* > 0.05) in the DAG content, while free fatty acids (FFA) decreased significantly (*p* < 0.05) as the heating time increased from 0 to 24 h. DAG was the predominant polar fraction found in RBDPO. DAG has both a hydrophilic group and a hydrophobic hydrocarbon that decrease the surface tension of the oil. Subsequently, oxygen can easily diffuse into the oil and accelerate the rate of oil oxidation [23]. However, the high temperature in this study limited oxygen solubility in oil. In addition, as no food was involved in this controlled heating study, the hydrolysis reaction was limited. FFAs have been found to be very volatile at high temperatures [24]. The decrease in FFA could be due to its evaporation during the heating treatment.

### 3.4. Effect of Temperature and Heating Time on the Oxidative Stability of RBDPO

A certain amount of energy is needed to trigger reactions that threaten the oxidative stability of the oil. At frying temperature, the energy requirement is more than fulfilled to break the bonds, e.g., the carbon–hydrogen bond on carbon 11 of linoleic acid and the oxygen–oxygen bond of alkyl hydroperoxide [25]. The peroxide value (PV) measures the content of hydroperoxides (the primary oxidation product) formed in the oil. Thus, PV is used as an oxidative index to provide the initial evidence of rancidity in fats and oils [26]. In this study, PV increased significantly (*p* < 0.05) after four hours of heating and decreased insignificantly (*p* > 0.05) as the heating time increased at all three heating temperatures (Table 3). A previous study showed that an elevated temperature enhanced the thermal degradation of the primary oxidation product, alkyl hydroperoxides [25]. These compounds are extremely unstable and readily decompose via fission, dehydration, and the formation of free radicals to yield a series of secondary oxidation products, especially when exposed at elevated temperatures [27,28]. 

On the other hand, the *p*-anisidine value (AV) measures the secondary oxidation products formed during the thermal treatment of oil, such as aldehydes and ketones [29]. Among these volatile compounds formed, aldehyde has been found to be the most abundant [19]. In the present work, the AV in fresh oil was 2.20, which indicates that the oil was of acceptable quality. A previous study reported that the AV of fresh oil should be less than 4.0–6.0 [30]. The AV increased significantly (*p* < 0.05) during the prolonged heating experiment for 24 h at all three selected heating temperatures. The increase in AV was attributed to the decomposition of hydroperoxides at all three heating temperatures, whereby the decomposition rate is higher than the formation rate [19]. A previous study demonstrated that a higher AV reduces the smoke point of an oil, which results in a poorer oil quality [19].

The total oxidation (TOTOX) value provides a better indication of the overall oxidative deterioration of fats and oils over time because it measures both primary and secondary products [28,30]. According to Table 3, the TOTOX value increased significantly (*p* < 0.05) with heating time and temperature. However, from 12 to 24 h of heating at 160 °C, 20 to 24 h of heating at 170 °C, and 16 to 24 h of heating at 180 °C, there were no significant (*p* > 0.05) increases in the TOTOX value. In fact, the lower the TOTOX value, the better the quality of oil. The increment in TOTOX value indicated that the quality of RBDPO had progressively degraded as the heating time and temperature increased.

### 3.5. Effect of Temperature and Heating Time on the Fatty Acid Composition of RBDPO

Fatty acid composition in oil is one of the most important parameters in determining its oxidative stability [31]. In general, it is known that oils that are more unsaturated oxidize more readily than those that are less unsaturated [32]. The higher the number of double bonds that exist in a fatty acid, the higher the rate and accumulation of primary oxidation compounds [25]. Table 4 shows the fatty acid composition changes in RBDPO at different heating times and temperatures. Palmitic acid (C16:0) and oleic acid (C18:1) are the predominant fatty acids found in RBDPO, followed by linoleic acid (C18:2), stearic acid (C18:0), as well as a minimal amount of lauric acid (C12:0) and myristic acid (C14:0) [33]. 

According to the literature, caprylic acid should not exist in fresh RBDPO. In our study, caprylic acid was not observed in fresh RBDPO but was found to increase significantly (*p* < 0.05) with heating time and temperature. The highest concentration (29 ppm) of caprylic acid was observed when RBDPO was heated for 24 h at 180 °C, which was approximately three times higher than RBDPO heated for 24 h at 160 °C. A previous study reported that oleic and linoleic acids are the major unsaturated fatty acids that are responsible for the formation of caprylic acid from their 9-hydroperoxides [8]. As volatile compounds easily escaped from the oil during heat treatment, the formation of caprylic acid, which is a more stable, non-volatile compound, indirectly measured the extent of oxidation. 

Linoleic acid decreased significantly (*p* < 0.05) after the heating study at 180 °C. In contrast, this led to an increase in saturated fatty acids such as stearic acid, because the fatty acid composition was determined based on the area normalization method. As linoleic acid has two double bonds, it is normal that it is extremely susceptible to degradation at a high temperature. A previous study also showed that a reduction of linoleic acid might contribute to an increase in palmitic acid [15]. The oxidation of linoleic acid plays a vital role in the formation of polar compounds in RBDPO [31].

The ratio of C18:2/C16:0 is used as an indicator to evaluate the oxidative deterioration status in oil. This is because palmitic acid is more stable towards oxidation, while linoleic acid is more susceptible to oxidation [15]. In this study, the ratio of C18:2/C16:0 decreased by 42.31% and 53.85% in RBDPO after being heated for 24 h at 160 and 180 °C, respectively. This indicated that the oxidation process progressed rapidly in RBDPO as the heating time and temperature increased.

### 3.6. Correlation among Analytical Parameters

Table 5 shows the correlation matrix of a few analytical parameters portraying the state of oil degradation upon heating. The correlation coefficients were determined from all the results of oil stability tests after the RBDPO was heated for 24 h at 160, 170, and 180 °C. The correlation study showed that there was a strong positive correlation among TPC, PTG, and oxTAG. These findings are in accordance with the study conducted by Gertz et al. (2014) on high oleic sunflower oil, linseed oil, sunflower oil, hydrogenated groundnut oil, and rapeseed oil. Therefore, the relationship demonstrates that the formation of PTG and oxTAG is sufficient to characterize the complete oil degradation process. In addition, TPC, PTG, and oxTAG also showed a strong positive correlation with epoxy, keto, and hydroxy acid formation. On the contrary, TPC, PTG, and oxTAG demonstrated a strong negative correlation with DAG and FFA. A weak negative correlation was found between TPC, PTG, oxTAG, epoxy, keto, and hydroxy acids with PV. The weak correlation indicated that PV might not be a suitable parameter to assess the quality of used oil, as it only measured the primary oxidation products. TPC, PTG, oxTAG, epoxy, keto, and hydroxy acids showed a stronger positive correlation with AV, TOTOX, and caprylic acid formation. The strong correlation again proved that caprylic acid formation could be proposed as one a marker to evaluate the extent of polymerization and oxidation that takes place in oil during thermal treatment.

## 4. Conclusions

The heating time and temperature had significant effects on the formation of oxTAG, TPC, and PTG. The oxTAG, TPC, and PTG concentrations significantly increased (*p* < 0.05) with heating time and temperature. The most abundant changes in RBDPO quality were observed during heating at the highest temperature (180 °C). The strong positive correlation among these parameters indicated that these parameters are suitable to illustrate the oil degradation extent in controlled heating studies or frying applications. The results of this study provide a complete picture regarding the oxidative and structural degradation in palm olein at elevated temperatures (in a controlled heating system without food).

## Figures and Tables

**Table 1:** Epoxy fatty acid methyl esters (FAMEs), ketoFAMEs and hydroxyFAMEs (g/100g) in palm olein heated at different temperatures for different times.

Heating Temperature (°C)	Heating Time (h)	EpoxyFAMEs	KetoFAMEs	HydroxyFAMEs	Total
Fresh oil	0	0.00 ± 0.00 ^a^	0.00 ± 0.00 ^a^	0.00 ± 0.00 ^a^	0.00 ± 0.00 ^a^
160	4	0.05 ± 0.00 ^ab^	0.05 ± 0.01 ^ab^	0.13 ± 0.01 ^bc^	0.23 ± 0.05 ^ab^
	8	0.11 ± 0.01 ^abc^	0.08 ± 0.02 ^ab^	0.19 ± 0.02 ^bcd^	0.39 ± 0.06 ^bcdef^
	12	0.17 ± 0.02 ^cde^	0.12 ± 0.03 ^abc^	0.21 ± 0.07 ^bcde^	0.51 ± 0.05 ^bcdefg^
	16	0.27 ± 0.01 ^ef^	0.20 ± 0.03 ^bcde^	0.29 ± 0.02 ^def^	0.75 ± 0.05 ^fghi^
	20	0.36 ± 0.03 ^fgh^	0.21 ± 0.06 ^bcde^	0.27 ± 0.04 ^def^	0.83 ± 0.07 ^ghi^
	24	0.42 ± 0.07 ^gh^	0.21 ± 0.08 ^bcde^	0.25 ± 0.08 ^cdef^	0.88 ± 0.11 ^hi^
170	4	0.13 ± 0.01^bcd^	0.05 ± 0.01^ab^	0.13 ± 0.01^bc^	0.31 ± 0.05^abcd^
	8	0.18 ± 0.03 ^cde^	0.11 ± 0.03 ^ab^	0.18 ± 0.03 ^bcd^	0.46 ± 0.04 ^bcdef^
	12	0.24 ± 0.03 ^def^	0.18 ± 0.02 ^bcd^	0.23 ± 0.02 ^bcdef^	0.66 ± 0.03 ^defgh^
	16	0.32 ± 0.10 ^fg^	0.28 ± 0.13 ^cdef^	0.29 ± 0.08 ^def^	0.90 ± 0.02 ^hi^
	20	0.31 ± 0.09 ^fg^	0.29 ± 0.10 ^def^	0.28 ± 0.07 ^def^	0.88 ± 0.01 ^hi^
	24	0.36 ± 0.06 ^fgh^	0.35 ± 0.08 ^ef^	0.32 ± 0.05 ^ef^	1.03 ± 0.02 ^i^
180	4	0.09 ± 0.00 ^abc^	0.08 ± 0.01 ^ab^	0.10 ± 0.00 ^ab^	0.27 ± 0.01 ^abc^
	8	0.17 ± 0.02 ^cde^	0.08 ± 0.03 ^ab^	0.10 ± 0.02 ^ab^	0.36 ± 0.05 ^abcde^
	12	0.27 ± 0.02 ^ef^	0.17 ± 0.06 ^bcd^	0.17 ± 0.04 ^bcd^	0.61 ± 0.06 ^cdefgh^
	16	0.33 ± 0.02 ^fg^	0.19 ± 0.08 ^bcde^	0.19 ± 0.06 ^bcd^	0.71 ± 0.08 ^efghi^
	20	0.46 ± 0.06 ^h^	0.31 ± 0.06 ^def^	0.28 ± 0.04 ^def^	1.05 ± 0.16 ^i^
	24	0.73 ± 0.05 ^i^	0.42 ± 0.12 ^f^	0.35 ± 0.09 ^f^	1.50 ± 0.25 ^j^

Data are expressed as the mean ± standard deviation (*n* = 4). Mean values with different superscripts in the same column are significantly different at *p* < 0.05.

**Table 2:** Changes in the polar fraction content and composition in palm olein heated at different temperatures for different times.

Heating Temperature (°C)	Heating Time (h)	Polar Fraction Content (%, g 100 g^−1^)	Polar Fraction Composition (%, g 100 g^−1^)				
			TAG Oligomers	TAG Dimers	Oxidized TAG Monomers	Diacylglycerols	Free Fatty Acids
Fresh oil	0	8.38 ± 0.53 ^a^	0.00 ± 0.00 ^a^	0.27 ± 0.05 ^a^	1.21 ± 0.05 ^a^	6.54 ± 0.59 ^a^	0.35 ± 0.11 ^c^
160	4	10.85 ± 0.42 ^ab^	0.25 ± 0.06 ^a^	1.67 ± 0.17 ^ab^	2.49 ± 0.28 ^ab^	6.17 ± 0.65 ^a^	0.27 ± 0.04 ^bc^
	8	13.47 ± 0.45 ^bc^	0.75 ± 0.04 ^ab^	2.73 ± 0.19 ^bcd^	3.53 ± 0.17 ^bcd^	6.23 ± 0.22 ^a^	0.24 ± 0.04 ^bc^
	12	16.00 ± 1.31 ^cd^	1.34 ± 0.15 ^abcd^	3.65 ± 0.33 ^de^	4.91 ± 0.33 ^de^	5.91 ± 0.79 ^a^	0.19 ± 0.03 ^abc^
	16	19.15 ± 0.28 ^de^	1.98 ± 0.06 ^bcde^	4.80 ± 0.11 ^ef^	6.05 ± 0.25 ^ef^	6.15 ± 0.15 ^a^	0.18 ± 0.07 ^abc^
	20	22.11 ± 0.41 ^efg^	2.76 ± 0.22 ^defg^	5.75 ± 0.11 ^fgh^	7.43 ± 0.18 ^fg^	6.08 ± 0.15 ^a^	0.09 ± 0.10 ^ab^
	24	25.05 ± 0.30 ^fghi^	3.64 ± 0.30 ^fg^	6.63 ± 0.06 ^ghi^	8.61 ± 0.24 ^gh^	6.05 ± 0.27 ^a^	0.13 ± 0.15 ^abc^
170	4	12.04 ± 0.57 ^abc^	0.42 ± 0.24 ^a^	1.98 ± 0.36 ^abc^	3.10 ± 0.24 ^abcd^	6.27 ± 0.47 ^a^	0.25 ± 0.05 ^bc^
	8	15.95 ± 1.20 ^cd^	1.25 ± 0.31 ^abc^	3.43 ± 0.59 ^cde^	4.80 ± 0.44 ^cde^	6.24 ± 0.38 ^a^	0.22 ± 0.15 ^abc^
	12	19.95 ± 2.09 ^de^	2.04 ± 0.47 ^bcde^	4.81 ± 0.79 ^ef^	6.68 ± 1.04 ^efg^	6.23 ± 0.31 ^a^	0.19 ± 0.07 ^abc^
	16	23.36 ± 2.56 ^efgh^	2.84 ± 0.64 ^efg^	5.99 ± 1.07 ^fgh^	8.02 ± 1.09 ^fgh^	6.37 ± 0.09 ^a^	0.14 ± 0.11 ^abc^
	20	27.46 ± 3.33 ^hij^	4.19 ± 1.02 ^gh^	7.09 ± 1.21 ^hi^	9.90 ± 1.25 ^hi^	6.21 ± 0.11 ^a^	0.06 ± 0.12 ^ab^
	24	30.35 ± 3.21 ^jk^	5.40 ± 0.86 ^hi^	7.89 ± 1.08 ^ij^	10.96 ± 1.42 ^i^	6.10 ± 0.17 ^a^	0.00 ± 0.00 ^a^
180	4	12.14 ± 1.22 ^abc^	0.46 ± 0.20 ^a^	2.42 ± 0.53 ^bcd^	2.86 ± 0.52 ^abc^	6.19 ± 0.12 ^a^	0.21 ± 0.09 ^abc^
	8	15.92 ± 1.68 ^cd^	1.44 ± 0.09 ^abcde^	3.84 ± 0.69 ^de^	4.84 ± 0.68 ^cde^	5.68 ± 0.68 ^a^	0.13 ± 0.02 ^abc^
	12	21.19 ± 1.96 ^ef^	2.68 ± 0.60 ^cdef^	5.45 ± 0.71 ^fg^	6.81 ± 0.96 ^efg^	6.16 ± 0.38 ^a^	0.09 ± 0.10 ^ab^
	16	25.87 ± 2.37 ^ghij^	3.84 ± 0.79 ^fg^	6.95 ± 0.58 ^ghi^	8.65 ± 1.37 ^gh^	6.19 ± 0.38 ^a^	0.24 ± 0.06 ^bc^
	20	29.25 ± 2.12 ^ijk^	5.30 ± 0.80 ^hi^	7.82 ± 0.73 ^ij^	9.93 ± 0.89 ^hi^	6.20 ± 0.35 ^a^	0.00 ± 0.00 ^a^
	24	32.92 ± 1.96 ^k^	6.74 ± 1.27 ^i^	8.91 ± 0.43 ^j^	11.18 ± 0.95 ^i^	6.08 ± 0.55 ^a^	0.00 ± 0.00 ^a^

Data are expressed as the mean ± standard deviation (*n* = 4). Mean values with different superscripts in the same column are significantly different at *p* < 0.05.

**Table 3:** Changes in the peroxide value, anisidine value and total oxidation (TOTOX) value in palm olein heated at different temperatures for different times.

Heating Temperature (° C)	Heating Time (h)	Peroxide Value (meq O_2_/kg)	Anisidine Value	TOTOX Value
Fresh oil	0	1.40 ± 0.22 ^a^	2.20 ± 0.64 ^a^	5.00 ± 0.96 ^a^
160	4	8.12 ± 1.89 ^bc^	38.87 ± 1.08 ^b^	55.10 ± 4.84 ^bc^
	8	7.00 ± 1.48 ^bc^	53.82 ± 6.07 ^cde^	67.81 ± 5.40 ^cdef^
	12	8.12 ± 3.68 ^bc^	58.57 ± 5.44 ^cdef^	74.82 ± 3.01 ^defg^
	16	6.75 ± 1.19 ^bc^	66.53 ± 4.59 ^efg^	80.02 ± 5.52 ^fghi^
	20	6.87 ± 1.38 ^bc^	67.44 ± 5.72 ^efg^	81.18 ± 4.01 ^ghi^
	24	6.13 ± 1.97 ^b^	70.44 ± 7.65 ^fgh^	82.69 ± 6.63 ^ghi^
170	4	8.75 ± 2.96 ^bc^	34.01 ± 3.88 ^b^	51.50 ± 4.29 ^b^
	8	7.87 ± 1.89 ^bc^	47.79 ± 5.07 ^bcd^	63.52 ± 4.76 ^bcd^
	12	7.50 ± 0.58 ^bc^	64.32 ± 13.25 ^efg^	79.31 ± 12.18 ^fgh^
	16	7.87 ± 0.75 ^bc^	58.83 ± 6.75 ^def^	74.58 ± 7.45 ^defg^
	20	8.62 ± 0.74 ^bc^	68.83 ± 1.37 ^fg^	86.07 ± 1.67 ^ghi^
	24	7.25 ± 0.65 ^bc^	76.07 ± 8.87 ^gh^	90.56 ± 7.58 ^hij^
180	4	10.10 ± 0.47 ^c^	44.64 ± 2.68 ^bc^	64.84 ± 3.07 ^cde^
	8	9.74 ± 0.30 ^bc^	58.36 ± 2.99 ^cdef^	77.83 ± 3.08 ^efgh^
	12	9.61 ± 0.31 ^bc^	73.74 ± 0.68 ^gh^	92.96 ± 0.87 ^ij^
	16	8.36 ± 0.25 ^bc^	84.24 ± 0.74 ^hi^	100.95 ± 0.53 ^jk^
	20	7.99 ± 0.36 ^bc^	93.62 ± 2.43 ^i^	109.59 ± 2.45 ^k^
	24	7.49 ± 0.58 ^bc^	96.33 ± 2.48 ^i^	111.31 ± 3.07 ^k^

Data are expressed as the mean ± standard deviation (*n* = 4). Mean values with different superscripts in the same column are significantly different at *p* < 0.05.

**Table 4:** Changes in the fatty acid composition in palm olein heated at different temperatures for different times.

Heating Temperature (°C)	Heating Time (h)	Fatty Acid Composition (Relative Percentages)								C8 (ppm)
		C12:0	C14:0	C16:0	C18:0	C18:1	C18:2	C18:3	C18:2/C16:0	
Fresh oil	0	0.24 ± 0.00 ^a^	1.53 ± 0.04^c^	39.80 ± 0.01 ^a^	4.19 ± 0.00 ^a^	43.90 ± 0.05 ^b^	10.33 ± 0.01 ^cde^	N.D.	0.26 ± 0.00 ^i^	0.00 ± 0.00 ^a^
160	4	0.21 ± 0.02 ^a^	1.03 ± 0.07 ^ab^	41.43 ± 0.62 ^a^	7.69 ± 0.82 ^ab^	39.21 ± 1.42 ^ab^	10.43 ± 0.73 ^cde^	N.D.	0.25 ± 0.0 ^hi^	0.00 ± 0.00 ^a^
	8	0.22 ± 0.01 ^a^	0.98 ± 0.05 ^ab^	41.40 ± 0.93 ^a^	9.92 ± 0.10 ^bc^	38.14 ± 1.53 ^ab^	9.26 ± 0.50 ^bcde^	N.D.	0.22 ± 0.01 ^fghi^	2.84 ± 0.50 ^ab^
	12	0.23 ± 0.01 ^a^	1.02 ± 0.05 ^ab^	41.88 ± 1.77 ^a^	9.82 ± 0.24 ^bc^	38.20 ± 1.31 ^ab^	8.76 ± 0.51 ^bcde^	N.D.	0.21 ± 0.02 ^defghi^	4.83 ± 0.43 ^abcd^
	16	0.23 ± 0.02 ^a^	1.10 ± 0.22 ^ab^	42.08 ± 3.41 ^a^	11.58 ± 0.87 ^c^	36.87 ± 3.07 ^ab^	8.02 ± 1.05 ^abcd^	N.D.	0.19 ± 0.04 ^bcdefg^	5.78 ± 0.32 ^bcd^
	20	0.23 ± 0.02 ^a^	1.02 ± 0.04 ^ab^	42.98 ± 0.81 ^a^	12.48 ± 0.74 ^c^	35.27 ± 1.14 ^ab^	7.89 ± 0.44 ^abc^	N.D.	0.18 ± 0.01 ^bcdef^	7.07 ± 0.56 ^bcd^
	24	0.23 ± 0.01 ^a^	1.08 ± 0.11 ^ab^	45.84 ± 4.31 ^a^	12.53 ± 0.96 ^c^	33.14 ± 3.92 ^a^	7.03 ± 0.25 ^ab^	N.D.	0.15 ± 0.02 ^abc^	9.69 ± 1.83 ^de^
170	4	0.23 ± 0.02 ^a^	1.08 ± 0.10 ^ab^	42.46 ± 0.75 ^a^	5.18 ± 0.28 ^a^	40.35 ± 1.85 ^ab^	10.70 ± 1.00 ^e^	N.D.	0.25 ± 0.02 ^hi^	0.00 ± 0.00 ^a^
	8	0.25 ± 0.03 ^a^	1.17 ± 0.17 ^abc^	43.65 ± 1.29 ^a^	5.33 ± 0.44 ^a^	39.02 ± 2.14 ^ab^	10.44 ± 1.75 ^de^	N.D.	0.24 ± 0.04 ^ghi^	3.93 ± 0.32 ^abc^
	12	0.24 ± 0.02 ^a^	1.20 ± 0.16 ^abc^	45.69 ± 1.97 ^a^	6.49 ± 0.24 ^ab^	36.95 ± 1.68 ^ab^	9.28 ± 0.16 ^bcde^	N.D.	0.20 ± 0.01 ^cdefgh^	5.27 ± 0.58 ^abcd^
	16	0.24 ± 0.03 ^a^	1.11 ± 0.07 ^ab^	45.24 2.38 ^a^	6.77 ± 2.60 ^ab^	38.08 ± 0.82 ^ab^	8.44 ± 0.69 ^abcde^	N.D.	0.19 ± 0.01 ^bcdefg^	13.49 ± 0.91 ^ef^
	20	0.26 ± 0.09 ^a^	1.16 ± 0.36 ^abc^	47.69 ± 13.55 ^a^	6.81 ± 3.55 ^ab^	36.71 ± 11.76 ^ab^	7.21 ± 1.52 ^ab^	N.D.	0.16 ± 0.03 ^abcd^	13.82 ± 1.51 ^ef^
	24	0.26 ± 0.04 ^a^	1.20 ± 0.15 ^abc^	49.09 ± 5.71 ^a^	7.32 ± 1.51 ^ab^	34.67 ± 5.12 ^ab^	7.29 ± 0.89 ^ab^	N.D.	0.15 ± 0.01 ^abc^	16.77 ± 0.79 ^fg^
180	4	0.19 ± 0.05 ^a^	0.93 ± 0.13 ^a^	43.94 ± 5.40 ^a^	5.02 ± 1.50 ^a^	39.28 ± 5.89 ^ab^	10.56 ± 1.47 ^de^	N.D.	0.24 ± 0.00 ^ghi^	2.16 ± 0.29 ^ab^
	8	0.24 ± 0.05 ^a^	1.11 ± 0.17 ^ab^	44.94 ± 4.20 ^a^	5.30 ± 3.16 ^a^	38.32 ± 3.89 ^ab^	9.94 ± 1.35 ^cde^	N.D.	0.22 ± 0.02 ^efghi^	5.05 ± 1.98 ^abcd^
	12	0.22 ± 0.01 ^a^	1.10 ± 0.05 ^ab^	46.58 ± 2.33 ^a^	5.52 ± 1.33 ^a^	37.57 ± 2.61 ^ab^	8.68 ± 0.81 ^bcde^	N.D.	0.19 ± 0.01 ^bcdefg^	9.00 ± 3.25 ^cde^
	16	0.23 ± 0.02 ^a^	1.15 ± 0.07 ^ab^	48.37 ± 2.77 ^a^	5.82 ± 0.43 ^a^	35.87 ± 3.32 ^ab^	8.14 ± 0.88 ^abcd^	N.D.	0.17 ± 0.01 ^abcde^	19.29 ± 6.12 ^g^
	20	0.22 ± 0.01 ^a^	1.17 ± 0.07 ^abc^	49.26 ± 2.60 ^a^	5.94 ± 1.01 ^a^	35.63 ± 2.11 ^ab^	7.18 ± 0.41 ^ab^	N.D.	0.15 ± 0.01 ^ab^	21.08 ± 2.15 ^g^
	24	0.24 ± 0.01 ^a^	1.34 ± 0.20^bc^	50.50 ± 4.33 ^a^	6.12 ± 0.75 ^ab^	34.96 ± 1.86 ^ab^	6.11 ± 1.57 ^a^	N.D.	0.12 ± 0.04 ^a^	29.13 ± 3.87 ^h^

N.D.: Not detected; Data are expressed as the mean ± standard deviation (*n* = 4). Mean values with different superscripts in the same column are significantly different at *p* < 0.05 in RBDPO after being heated for 24 h at 160 and 180 °C. This indicates that the oxidation process progressed rapidly in RBDPO as the heating time and temperature increased.

**Table 5:** Correlation coefficients (*r*) for analytical parameters.

	TPC	TAGO	TAGD	OXTAGM	DAG	FFA	EPOXY	KETO	HYDROXY	PV	AV	TOTOX	C8
TPC	1.000	-	-	-	-	-	-	-	-	-	-	-	-
TAGO	0.976	1.000	-	-	-	-	-	-	-	-	-	-	-
TAGD	0.792	0.838	1.000	-	-	-	-	-	-	-	-	-	-
OXTAGM	0.845	0.876	0.950	1.000	-	-	-	-	-	-	-	-	-
DAG	−0.900	−0.935	−0.969	−0.980	1.000	-	-	-	-	-	-	-	-
FFA	−0.786	−0.815	−0.878	−0.874	0.874	1.000	-	-	-	-	-	-	-
EPOXY	0.886	0.893	0.703	0.714	−0.793	−0.710	1.000	-	-	-	-	-	-
KETO	0.816	0.811	0.651	0.690	−0.739	−0.661	0.865	1.000	-	-	-	-	-
HYDROXY	0.750	0.752	0.677	0.719	−0.738	−0.701	0.802	0.886	1.000	-	-	-	-
PV	−0.472	−0.485	−0.634	−0.658	0.610	0.676	−0.440	−0.409	−0.588	1.000	-	-	-
AV	0.853	0.897	0.909	0.880	−0.928	−0.850	0.817	0.752	0.722	−0.644	1.000	-	-
TOTOX	0.858	0.905	0.886	0.848	−0.913	−0.810	0.824	0.757	0.685	−0.507	0.986	1.000	-
C8	0.885	0.865	0.623	0.652	−0.735	−0.628	0.870	0.816	0.672	−0.329	0.786	0.813	1.000

TPC: Total polar compounds; TAGO: Triacylglycerol oligomers; TAGD: Triacylglycerol dimers; OXTAGM: Oxidized triacylglycerol monomers; DAG: Diacylglycerols; FFA: Free fatty acids; EPOXY: Epoxy fatty acids; KETO: Keto fatty acids; HYDROXY: Hydroxyl fatty acids; PV: Peroxide value; AV: *p*-anisidine value; TOTOX: Total oxidation value; and C8: Caprylic acid; Data are expressed as the mean ± standard deviation (*n* = 4). Mean values with different superscripts in the same column are significantly different at *p* < 0.05.
